# Fabrication and Characterization of a Magnetic Drilling Actuator for Navigation in a Three-dimensional Phantom Vascular Network

**DOI:** 10.1038/s41598-018-22110-5

**Published:** 2018-02-27

**Authors:** Sunkey Lee, Seungmin Lee, Sangwon Kim, Chang-Hwan Yoon, Hun-Jun Park, Jin-young Kim, Hongsoo Choi

**Affiliations:** 1 Department of Robotics Engineering, DGIST, Daegu, 42988 Republic of Korea; 2 DGIST-ETH Microrobot Research Center, DGIST, Daegu, 42988 Republic of Korea; 3 Institute of Robotics and Intelligent Systems, ETH Zurich, Zurich, CH-8092 Switzerland; 4 Cardiovascular Center & Department of Internal Medicine, Seoul National University Bundang Hospital, Seongnam, Gyeonggi 13620 Republic of Korea; 5 Cardiology Division, Department of Internal Medicine, College of Medicine, The Catholic University of Korea, Seoul, 06591 Republic of Korea

## Abstract

Intravascular microrobots have emerged as a promising tool for vascular diseases. They can be wirelessly and precisely manipulated with a high degree of freedom. Previous studies have evaluated their drilling performance and locomotion, and showed the feasibility of using microrobots for biomedical applications in two-dimensional space. However, it is critical to validate micro-drillers in a three-dimensional (3D) environment because gravity plays an important role in a 3D environment and significantly affects the performance of the micro-drillers in vascular networks. In this work, we fabricated magnetic drilling actuators (MDAs) and characterized their locomotion and drilling performance in vascular network-mimicking fluidic channels. The MDAs were precisely manipulated in the fluidic channel network in both horizontal and vertical planes, selecting and moving through the desired path via the junctions of multiple channels. The MDAs also accurately navigated an artificial thrombosis in an artificial 3D vascular network and successfully drilled through it. The results obtained here confirmed the precise manipulation and drilling performance of the developed MDAs in 3D. We think that the MDAs presented in this paper have great potential as intravascular drillers for precise thrombus treatment.

## Introduction

Vascular disease is any abnormal condition of the blood vessels, such as thrombosis^1^. Its incidence has increased in modern times due to intake of fatty, high-calorie foods; lack of exercise; and psychological stress^2,3^. Vascular disease can interfere with blood circulation, reduce organ function, and lead to a range of severe or fatal health problems irrespective of age or sex; e.g., stroke, peripheral artery disease (PAD), coronary artery disease (CAD), and varicose veins^4,5^. The mortality rate due to cardiovascular disorders, which can lead to cardiac infarction, has increased recently. The most common treatments for vascular diseases are drug therapy, vascular bypass graft, and percutaneous transluminal angioplasty (PTA). Drug therapy dissolves a thrombus using, for example, a thrombolytic agent or anticoagulant^6,7^. However, this may adversely affect the hemostatic response as thrombosis formation stops hemorrhaging of damaged blood vessels^8^. In vascular bypass grafting, the grafted blood vessel bypasses the occluded vessels^9^. However, this involves a cumbersome laparotomy procedure with a prolonged recovery and has side-effects. PTA is the most frequently used treatment for clogged blood vessels, particularly for chronic total occlusion (CTO) in the cardiovascular network, because it is a relatively simple procedure, minimally invasive, and has a short recovery time. A guide wire and catheter are used to penetrate the occlusion and a stent is inserted to expand the narrowed blood vessels^10,11^. However, the guide wire and catheter have few degrees of freedom (DOFs) in their movement and are manually navigated to the lesion through the vascular network by a surgeon. Therefore, surgeons must be highly skilled and experienced to perform PTA without damaging the blood vessel^12,13^.

Recently, intravascular microrobots show promise for treatment of vascular diseases and are expected to remedy the shortcomings of current modalities^14^. Their small size (micrometers to millimeters) makes the procedure relatively non-invasive^15–18^. In addition, microrobots can be wirelessly and precisely manipulated with a high degree of freedom (DOF) using an external magnetic field^19–25^. Park *et al*. developed the enhanced electromagnetic actuation (EMA) system, which combined Helmholtz and Maxwell coils, and evaluated its drilling performance using an artificial thrombosis comprising a 0.2% agarose gel^26^. Nam *et al*. tested various microrobot structures to optimize their drilling performance. They fabricated a microrobot that moves by a crawling motion, and characterized it to achieve efficient crawling and drilling performance in a vascular simulation channel^27^. A dual-body magnetic helical robot was also developed to drill a lesion model using both chemical and physical methods^28^. Additionally, a new magnetic minirobot with anchoring and drilling abilities was proposed^29^. These microrobots were demonstrated to be feasible for biomedical applications in two-dimensional (2D) space. The microrobots were manipulated in a thrombosis-containing single channel or Y-shaped channel in a horizontal plane. However, this is not representative of the *in vivo* situation; the vascular network is complex and three-dimensional (3D). It is critical to validate micro-drillers in a 3D environment because gravity affects their performance in the vascular network.

In the work described in this paper, we fabricated magnetic drilling actuators (MDAs) with different numbers of spirals to optimize their propulsion and drilling force in a fluidic environment by using a 3D printing process with a diametrically magnetized neodymium (Nd) alloy magnet. The MDAs were manipulated under a rotating magnetic field (RMF) generated from the EMA system and characterized for their locomotion and drilling performance in vascular-network-mimicking fluidic channels in both horizontal and vertical planes. To demonstrate their feasibility as intravascular drillers, particularly for CTO treatment, we assessed the navigation of MDAs at the junction of multiple channels and their drilling performance in a 3D phantom cardiovascular network with a thrombus.

## Results

### Design and fabrication of MDAs

**Figure 1:**
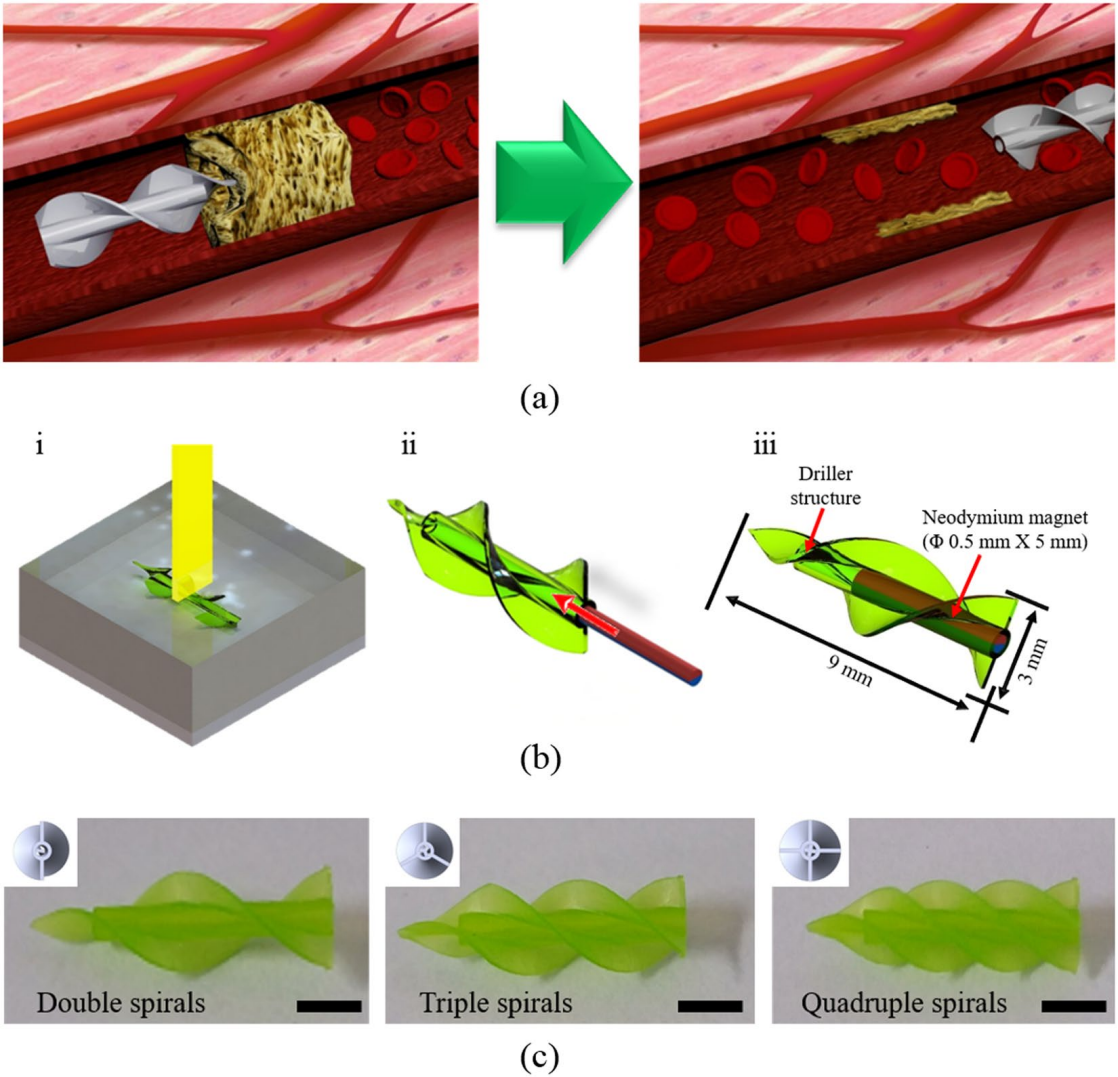
Design and fabrication of MDA. (**a**) Conceptual schematic of the MDA as an intravascular driller in a 3D vascular network. (**b**) Fabrication procedures of the MDA: (i) 3D printing of the MDA body, (ii) inserting Nd magnet into the MDA body, and (iii) dimensions of the assembled MDA. (**c**) Optical images of the fabricated MDAs with double, triple, and quadruple spirals. Scale bar represents 2 mm.

Figure 1a shows a conceptual schematic diagram of thrombosis treatment using the MDA in a complex vascular network in the body. After minimally invasive introduction into the body, the MDA is precisely navigated to a lesion in the vascular network by adjusting an external magnetic field; then, it begins to drill through the thrombus to open a pathway to recirculate the blood. The MDA has the possibility of being navigated around various environments, for instance, the circulatory, urinary, and central nervous systems, inside the human body where the conventional PTA methods such as guide-wire-based catheterization cannot reach, because the MDA is operated precisely and wirelessly from outside the body. The MDA was designed with a 3D CAD tool (Solidworks, Dassault Systèmes SolidWorks Corp., USA) and fabricated by 3D stereolithography (Proto Labs Inc., USA) (Fig. 1b). The MDA is spiral-shaped and of 3 mm in diameter and 9 mm in length, and has a cylindrical inner space in which a parylene-coated Nd magnet (0.5 mm diameter, 5 mm length), magnetized in the direction of the diameter, is inserted. MDAs were fabricated with double, triple, or quadruple spirals to investigate the effect on thrust force and determine the optimal number of spirals for propulsion and drilling performance in a 3D phantom vascular network (Fig. 1c).

### Magnetic manipulation of MDAs

The MDAs were controlled using an EMA system (Octomag, Aeon Scientific GmbH, Switzerland) with five degrees of freedom (DOFs) (Figure S1a). In this system, a magnetic field is generated by the linear superposition of individual fields from eight hemispherically configured electromagnetic coils carrying different currents^30^. The Nd magnet in the MDA was aligned with the magnetic field and then rotated with an RMF. During rotation, the MDA was able to swim forward by a corkscrewing motion. Therefore, the translation velocity can be controlled by adjusting the magnetic intensity and RMF frequency, and the swimming direction can be manipulated by changing the pivot of the RMF, which is controlled by the roll and pitch angle in the horizontal and vertical planes, respectively (Figure S1b).

**Figure 2:**
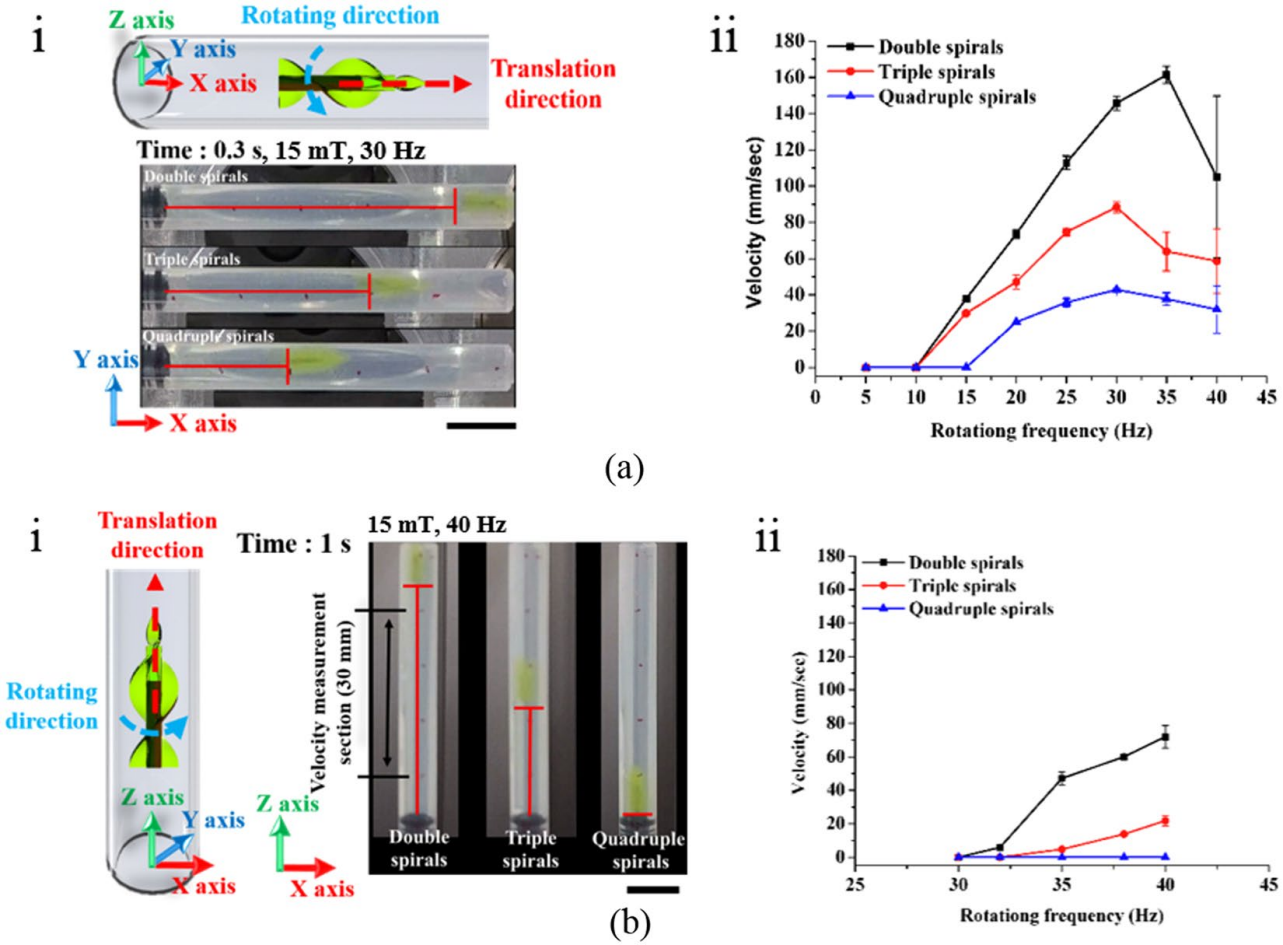
Velocities of MDAs in a single cylindrical channel. Translational velocity of the MDA with different numbers of spirals as a function of RMF frequency in a single cylindrical channel: (**a**) on the horizontal plane and (**b**) on the vertical plane. Scale bar represents 10 mm.

**Figure 3:**
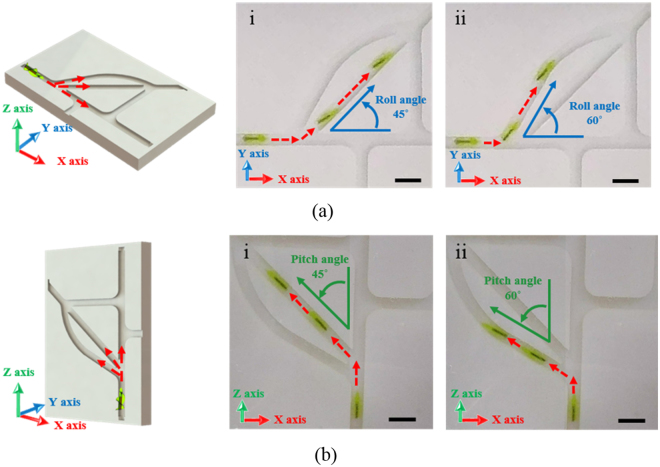
Manipulation of the MDA in 2D vascular network model. Translational locomotion of the MDA with double spirals in the 2D vascular network model placed: (**a**) on the horizontal plane and (**b**) on the vertical plane. (i) and (ii) show selective movement of the MDA toward 45° and 60° channels, respectively, by manipulation using EMA. Scale bar represents 10 mm. The MDA selected and moved along the desired path via the junctions of multiple channels with different angles.

In 3D space, the magnetic (*Fm*), resistive (*Fr*), and gravitational (*Fg*) forces affect MDA locomotion, which can be modeled by

1 $${F}_{m}+{F}_{r}+{F}_{g}={\rm{m}}\frac{{\rm{dv}}}{{\rm{dt}}}$$

where m is the mass and v is the translational velocity of the microrobot. To induce translational motion, *Fm* should be sufficient to overcome the other forces, *Fr* (surface friction and drag force) and *Fg*. While *Fg* does not change depending on the MDA position in the horizontal plane, it becomes critical when the MDA moves in the vertical plane. Therefore, it is important to characterize MDA locomotion in the vertical plane. We first measured the translational velocity of the MDA in a single cylindrical channel (4.5 mm internal diameter, 30 mm length) filled with distilled (DI) water and placed on a horizontal and vertical plane (Fig. 2a and b, Videos S1 and S2). Figure 2a-i presents schematic and optic images of translational locomotion of the MDA under a 15 mT, 30 Hz RMF. The MDA with double spirals was the fastest, moving approximately 50 mm in 0.3 sec. Figure 2b-i shows the translational velocity of the MDA as a function of RMF frequency. The RMF frequency was adjusted from 0 to 40 Hz with a magnetic intensity of 15 mT. The translational velocity in the horizontal plane of the MDA increased with increasing RMF frequency and decreasing number of spirals. In the case of double spirals, the translational velocity increased up to 161 mm/s at RMF of 35 Hz and then dramatically decreased and showed wobbling motion at RMF of >35 Hz. Thus, 35 Hz is the step-out frequency at which the applied magnetic torque is not sufficient to induce synchronous rotation of the MDA with the RMF^14,31,32^. The translational velocity in the vertical plane was also proportional to the RMF frequency and inversely proportional to the number of spirals (Fig. 2b-i and ii). The MDA with double spirals exhibited the highest velocity motion (maximum velocity, 72 mm/s at 40 Hz). More importantly, the translational velocity was generally lower in the vertical than the horizontal plane due to the gravitational force. An MDA with four spirals, unlike in the horizontal plane, did not show translational locomotion in the vertical plane. These results suggest that the gravitational force exerted a more marked effect on MDA locomotion in the vertical plane. It is important to characterize MDA manipulation in the vertical plane for use in 3D vascular networks. In consideration of the circulation of blood, manipulation of the MDA was characterized in a fluidic environment. The flow rate of the fluid was 30 mm/s and the characteristics of the MDA were evaluated in horizontal and vertical planes (inner diameter: 4 mm; Figure S2a). This fluidic environment was similar to the venous environment and showed the potential for manipulating the MDA in the vein^15^. We confirmed that the MDA overcomes the fluid flow at 15 mT with a 20 Hz RMF for the horizontal channel, and at 15 mT with a 40 Hz RMF for the vertical channel (Figure S2b and c, Videos S3 and S4). In addition to translational locomotion, the MDA must precisely navigate to a lesion in a complex phantom network, which involves selecting the desired path at the junction of multiple channels. Therefore, we also magnetically manipulated the MDA in a 2D fluidic channel network that contained junctions with 45° and 60° branches, as shown in Fig. 3. The channel was 4 mm in width and height, and was filled with DI water and then sealed with a transparent plastic cover. First, the MDA was actuated with a 90° pitch angle in a 10 mT magnetic field in the horizontal plane (Fig. 3a). As depicted in Fig. 3a-i and ii, application of an RMF with roll angles of 45 and 60° successfully guided the MDA through the 45° and 60° junctions, respectively (Video S5). Next, the MDA was selectively and precisely navigated through the 45° and 60° channels by adjusting the pitch angle from 0 to 45 (Fig. 3b-i) and 60° (Fig. 3b-ii), respectively, under a 15 mT RMF in the vertical plane (see also Video S6.).

### Drilling performance of MDAs

**Figure 4:**
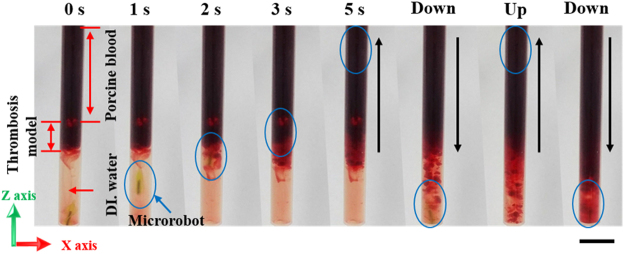
Drilling performance of MDA. Drilling performance of the MDA with an artificial thrombosis model in a single cylindrical tube on the vertical plane. Scale bar represents 10 mm.

Drilling by the MDAs in 2D and 3D was demonstrated using an RMF in a vertical tube (4.5 mm internal diameter) and a 3D cardiovascular network phantom, respectively, which were partially blocked by an artificial thrombosis. The thrombosis comprised 1.5% (w/w) gelatin and 98.5% (w/w) porcine blood and was formed as described in Figure S3. The 2D drilling test was conducted on the Z-axis only as this is more difficult than that along the X- or Y-axis due to the influence of gravity (Fig. 4). The tube was filled with 100% porcine blood and DI water, with a thrombosis in the center to mimic CTO in a blood vessel. After applying a 38 Hz RMF at 15 mT, the MDA rapidly approached and penetrated the thrombosis, reaching the top of the tube in ~4 s, overcoming friction and gravity. However, the porcine blood did not flow through the thrombosis because the MDA disrupted the thrombosis but did not create a passage sufficient for blood flow. A second drilling of the thrombosis increased the extent of penetration. After a second upward drilling, porcine blood flowed through the thrombosis and mixed with DI water (Video S7). Because the MDA is an independent intravascular driller and wirelessly controlled, simply changing the direction of the RMF enables drilling back and forth multiple times.

**Figure 5:**
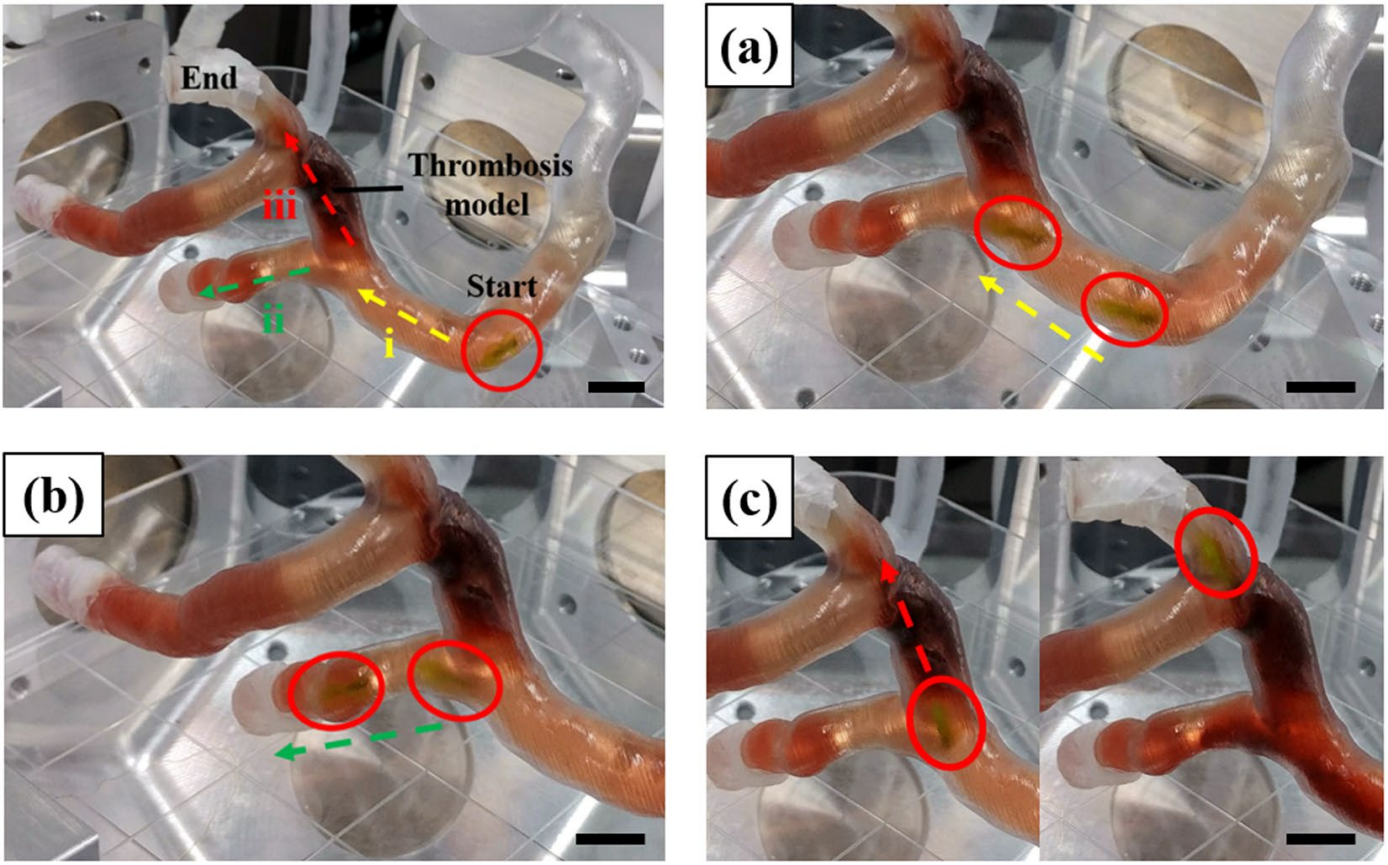
Magnetic manipulation and drilling performance in the 3D phantom. Magnetic manipulation and drilling performance of the MDA in the 3D phantom of coronary artery of the heart: (**a**) movement to a branch of the blood vessel, (**b**) selective movement through the lower blood vessel, and (**c**) selective movement to the blood vessel blocked by an artificial thrombosis model and perforation of it. Scale bar represents 10 mm.

Next, to validate its feasibility as an intravascular driller in a complex 3D vascular network, the MDA was manipulated in a 3D coronary artery phantom containing DI water and a thrombosis, as shown in Fig. 5 and Video S8. The phantom was placed in the workspace of the EMA system and a 20 Hz RMF at 15 mT was applied. First, the MDA was moved to the branch channel by specifying a roll angle of −20° and pitch angle of −70° (Fig. 5a), and selected and moved through the lower channel by adjusting the pitch to −100°, which demonstrates precise 3D navigation of the MDA at the junction of multiple channels (Fig. 5b). Next, the MDA was moved backward and began drilling the thrombosis in the upper channel with a roll of 0° and pitch of −20°. We used a 20 Hz RMF to move the MDA since it was too fast to control the MDA in a 3D phantom after the application of a 20 Hz RMF. The RMF was increased to 25 Hz for drilling of the thrombosis (Fig. 5c). During drilling, the disrupted blood clot was dislodged and the MDA penetrated the thrombosis in ~10 s (see Video S8). Therefore, the MDA can precisely navigate to a thrombosis in a complex 3D phantom vascular network under wireless magnetic control.

### Biocompatibility of MDAs

The biocompatibility of the MDA material was tested using human colorectal cancer (HCT116) cells, because MicroFine, an acrylonitrile butadiene styrene (ABS)-like material that was used to 3D-print the MDA structure, has not been investigated for biological samples. HCT116 cells were cultured with MDA material with or without a parylene coating in a 96-well plate for 1 day. Cell viability was measured by fluorescence imaging and compared with the control (HCT116 cells only without the MDA material). Figure S4a shows bright-field and fluorescence images of HCT116 cells after the 1-day cultivation. Blue fluorescence and red fluorescence indicate nuclei and dead cells, respectively. Cell viability was >95% in all experimental conditions, and did not differ significantly compared with the control, as shown in Figure S4b.

## Discussion

To use a magnetic actuator such as the MDA for blood vessel treatment, it is necessary to locate and visualize its position within the blood vessel. In the case of conventional vascular disease treatment, a contrast agent is injected into the blood vessel and the guidewire, catheter, and coronary artery may be visualized by X-ray or CT. This technique is known as angiography^33^. Intravascular ultrasound (IVUS) may also be used to obtain the location and orientation of the guidewire in a coronary artery^34^. The proposed MDA can be visualized by conventional angiography using X-ray or CT, as well as IVUS, and is also expected to be visualized using the *In Vivo* Imaging System (IVIS)^35–37^.

Different types of thrombi have various mechanical properties, from a soft jelly form to hard calcification. The guidewire and catheter used in coronary angiography are selected depending on the type of thrombosis. Choice of the appropriate guidewire is based on the characteristics of the thrombosis; therefore, different MDAs should be developed application to specific thrombi. However, the MDA may not be suitable for calcified plaque since it is not intended for the generation of high torques or grinding forces. Conventional CTO therapies use various scrupulous techniques such as antiplatelet therapy and vasodilators to prevent recurrence and complications after the procedure has been performed^38^. There is a risk of periprocedural myocardial infarction (rather than stroke) due to thrombosis because the application site is the coronary arteries. However, the size of debris caused by the drilling of the MDA is expected to be negligibly small compared to that caused by the large burr size (1.25~1.75 mm) used in rotational atherectomy (Rotablator). This is because the primary application of the proposed MDA is for soft thrombi. Also, general drug therapy (for example, antiplatelet and anticoagulant agents) may be helpful for the removal of debris from the soft thrombosis, both during and after procedure. However, further research should be conducted in consideration of the various possible side effects of the MDA, once we have made further more progress with respect to the engineering challenges of the actuator. After the procedure, the guidewire and catheter are retracted from the blood vessel. The proposed MDA can be also retracted after drilling of the thrombus by a catheter. In recent years, many studies have reported on biodegradable magnetic actuators for drug delivery^22,39^. The MDA could be developed to use the same kind of biodegradable materials, delivering anticoagulant drugs to prevent recurrence and complications after thrombus drilling.

Current procedures such as coronary angiography are performed using a guidewire and a catheter. Such procedures could damage vascular endothelial cells because they move along the wall of the vessel instead of the center to reach the desired position. If the MDA is used with a catheter, there may be no increase in vascular damage compared to existing procedures. Further research should be conducted to study the vascular damage caused by an artificial blood vessel with pulsation, or using an *in vivo* model. Efficient motion of MDAs, for example employing a corkscrewing motion, has already been implemented in a 2D environment which demonstrated their feasibility for biomedical applications. However, the drilling performance and manipulation of MDAs in a 3D environment have not been well reported upon. This may be due to difficulties in overcoming gravity to achieve sufficient propulsion and penetration force in a 3D environment. The aim of this research was to develop MDAs that can be precisely and wirelessly navigated, and that can perforate blockages in 3D vascular networks using external magnetic fields.

In terms of the biocompatibility of microrobots and MDAs, magnetic materials such as Ni and cobalt (Co) are coated and covered by titanium (Ti) or titanium oxide (TiO_2_), which do not have cytotoxicity for use *in vivo*. A Nd permanent magnet was chosen for our MDA due to its strong magnetic properties, which can improve drilling performance, overcoming the force of gravity via high propulsive force and torque under RMF. Nd magnets have higher remanence (B_r_: 0.6–1.3 T) and coercivity (H_ci_: 875–1990 kA/m) than other types of permanent magnet such as alnico (B_r_: 0.8–1.3 T, H_ci_: 51–129 kA/m) and ferrite (B_r_: 0.39 T, H_ci_: 239 kA/m) magnets^40–42^. However, Nd magnets are vulnerable to corrosion, which can result in disintegration of a magnet into small magnetic particles and severe deterioration. Therefore, parylene-coated Nd magnets were used for the developed MDA. Parylene is a biocompatible material that has been proven to improve biocompatibility in the medical and biotechnological fields^43–45^. In this work, MDAs with different numbers of spirals and an Nd magnet in the core were fabricated by 3D printing. Their locomotion in the horizontal and vertical planes was characterized using an EMA system, as vertical locomotion is critical for precisely manipulating MDAs in the vascular network *in vivo*. The MDA exhibited a corkscrewing forward motion in a RMF. The direction of translational locomotion was controlled by changing the roll and pitch angles of the RMF. Translational velocity in the horizontal and vertical planes increased with increasing RMF frequency and decreasing number of spirals. The translational velocity was generally lower in the vertical than the horizontal plane due to gravity. The MDA with double spirals showed the highest-velocity locomotion in both planes. For our MDA, the angle of spirals was fixed to 45°; however, the angle may affect the propulsion force, so it might require investigation of the translational velocity as a function of the spiral angles for further optimization. The MDA with double spirals could be precisely manipulated in a fluidic channel network in both the horizontal and vertical planes, which enables it to be guided through the desired path at the junction of multiple channels. We also demonstrated the drilling performance of the MDA in a 3D phantom coronary artery with a thrombosis. The MDA was accurately navigated to and penetrated the artificial thrombosis in an external RMF. Although the maximum translation velocity of the MDA was obtained with 35 Hz and 40 Hz RMF in a single cylindrical tube on the horizontal and vertical planes, respectively, lower RMF (20 Hz) was applied for manipulation in the 3D phantom coronary artery because it was too fast to control the external magnetic field manually. When penetrating the thrombosis model in the 3D phantom, the MDA was able to drill it with 25 Hz RMF, which did not exhibit upward movement against the force of gravity in the vertical cylindrical tube, because the angle of the artificial artery channel containing the thrombosis model was less than 90°. Pre-programmed magnetic control may improve rapid manipulation of the MDA in 3D complex vascular networks. For example, after the coordinates and map of the vascular network are obtained by pre-scanning prior to the magnetic manipulation, an optimized route can be calculated; then, the MDA can be automatically navigated to the lesion with prompt responses to rapid movement of the MDA. This may contribute to efficient manipulation of the MDA when it is applied in the human body. Regarding biocompatibility, the MDA material did not exert a cytotoxic effect. However, we may need to investigate long-term biocompatibility using biological samples to ensure that our MDA can be used in an *in vivo* environment. In conclusion, our results confirm that the proposed MDA can be precisely manipulated in 3D space and used to drill thrombi. The MDA presented herein has potential for removal of intravascular thrombi.

## Methods

### Magnetic manipulation of the MDA

The MDA was manipulated with 5 DOFs, namely, three translational (x, y and z) and two rotational (around the Z- and X-axes) DOFs, by an EMA system (Octomag, Aeon Scientific GmbH, Switzerland) (Figure S1a). The MDA was magnetically manipulated in the workspace among the electromagnetic coils and monitored by the camera on the top. Videos recorded at 15 frames per second were analyzed to characterize the translational locomotion, velocity, and drilling performance of the MDA. The direction of magnetization of the magnet in the MDA is parallel to that of the external magnetic field. When the magnetic field is rotated around the pivot, the MDA moves forward in a corkscrewing motion. Thus, the MDA translational direction was controlled by adjusting the angle of the pivot, which was itself altered by changing the roll angle in the horizontal plane (XY) (Figure S1b i) and the pitch angle in the vertical plane (XZ) (Figure S1b-ii).

### Fabrication of an artificial thrombosis model

To investigate the drilling performance of the MDA, an artificial thrombosis comprising gelatin and porcine blood was produced. Gelatin (1.5% w/w) was added to porcine blood (98.5% w/w), heated to 80 °C on a hot plate for 1 h, and cooled to 3 °C (Figure S2-a). Figure S2-b shows an optical image of the fabricated blood clot. The blood clot was inserted into a cylindrical tube and a 3D phantom coronary artery to mimic a thrombosis. In this manner, we fabricated an artificial thrombosis to verify the feasibility of the proposed MDA for CTO.

### Fluorescent staining of cells

Hoechst 33342 (blue) and propidium iodide (red) dye were mixed with medium at ratios of 1000:1 and 500:1, respectively, to stain nuclei and dead cells, and incubated for 10 min at 37 °C in 5% CO_2_. The ratio of dead cells was determined by analyzing red fluorescent pixels using a post-acquisition image-processing technique with Fiji/ImageJ (National Institutes of Health (NIH), USA), subtracted from 100%, and compared with the control.

## Electronic supplementary material

Supplementary Information

Video S1

Video S2

Video S3

Video S4

Video S5

Video S6

Video S7

Video S8
