# Supplementary material for: Fabrication and Characterization of a Magnetic Drilling Actuator for Navigation in a Three-dimensional Phantom Vascular Network
Source: Sci Rep. 2018 Feb 27;8:3691. doi: 10.1038/s41598-018-22110-5 (PMC5829245; doi:10.1038/s41598-018-22110-5)
Supplement: Supplementary file 1 — Supplementary Information [file 41598_2018_22110_MOESM1_ESM.doc]

**Supplementary Information**

**Manuscript title: Fabrication and Characterization of a Magnetic Drilling Actuator for Navigation in a Three-dimensional Phantom Vascular Network**

**Authors: Sunkey Lee, Seungmin Lee, Sangwon Kim, Chang-Hwan Yoon, Hun-Jun Park, Jin-young Kim and Hongsoo Choi**


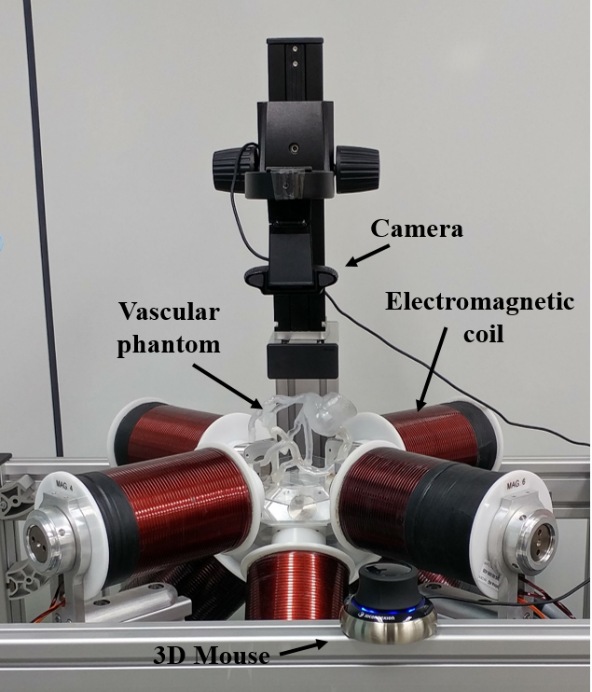

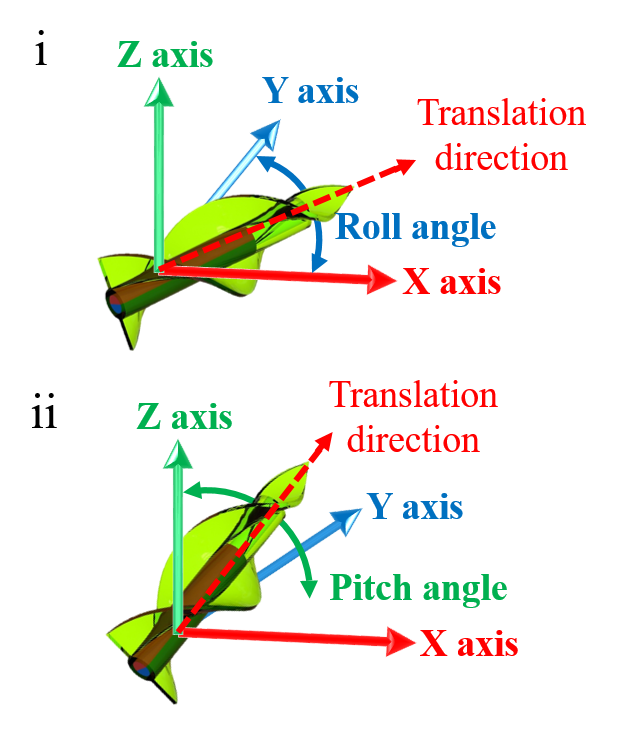


(a) (b)

**Figure S1.** Magnetic manipulation system and Conceptual schematic of the MDA manipulation. a) Experimental setup of the electromagnetic actuation (EMA) system and b) precise three-dimensional manipulation of the magnetic drilling actuator (MDA) in a rotating magnetic field (RMF) by changing i) the roll angle in the horizontal plane and ii) the pitch angle in the vertical plane.


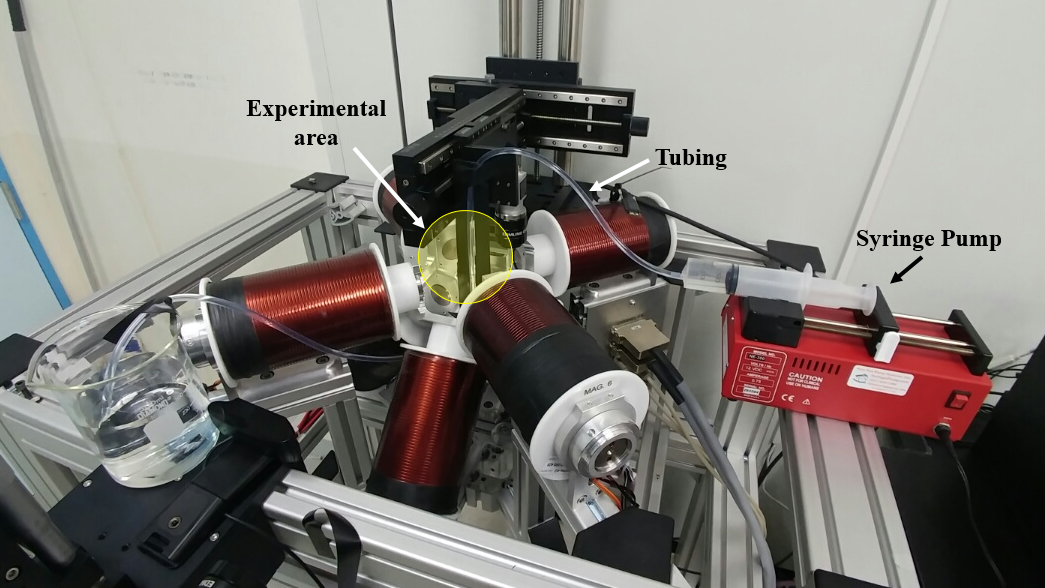


(a)


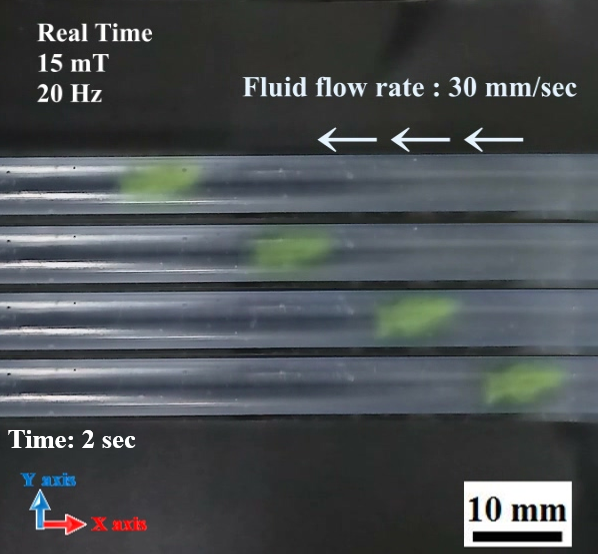

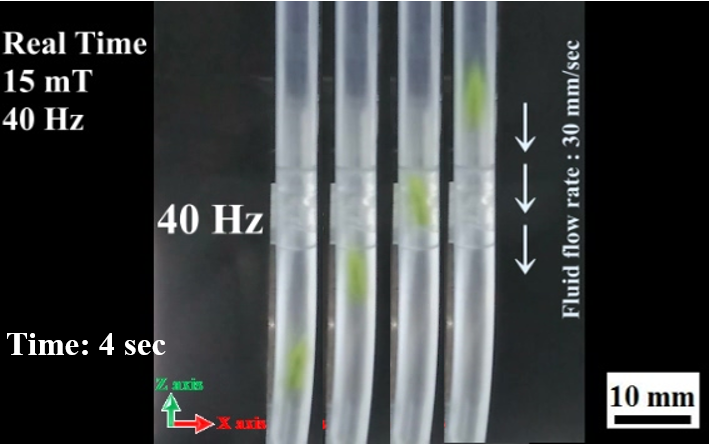


(b) (c)

**Figure S2.** Characteristics of MDA in fluidized fluid. a) Experimental setup, b) translational movement of the MDA in fluid flow in the horizontal plane and c) the vertical channels.


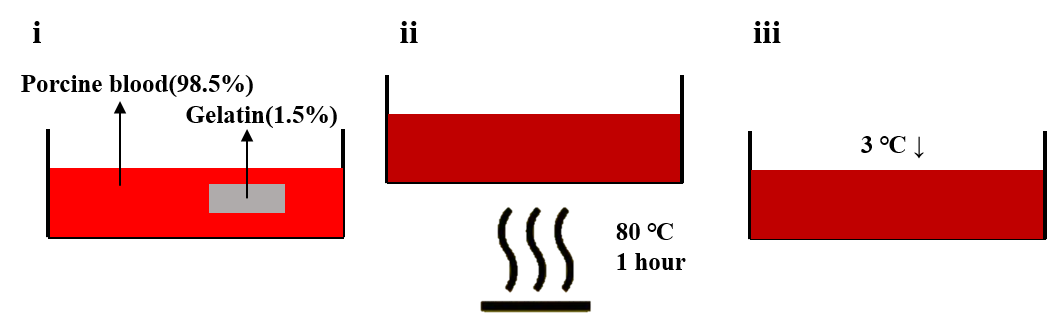

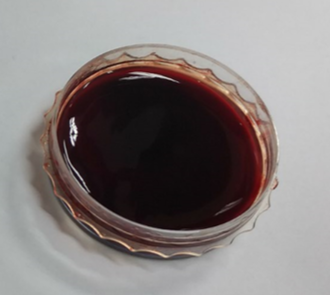


(a) (b)

**Figure S3. Fabrication process and result of thrombus.** a) Fabrication procedure and b) optical image of an artificial thrombus.


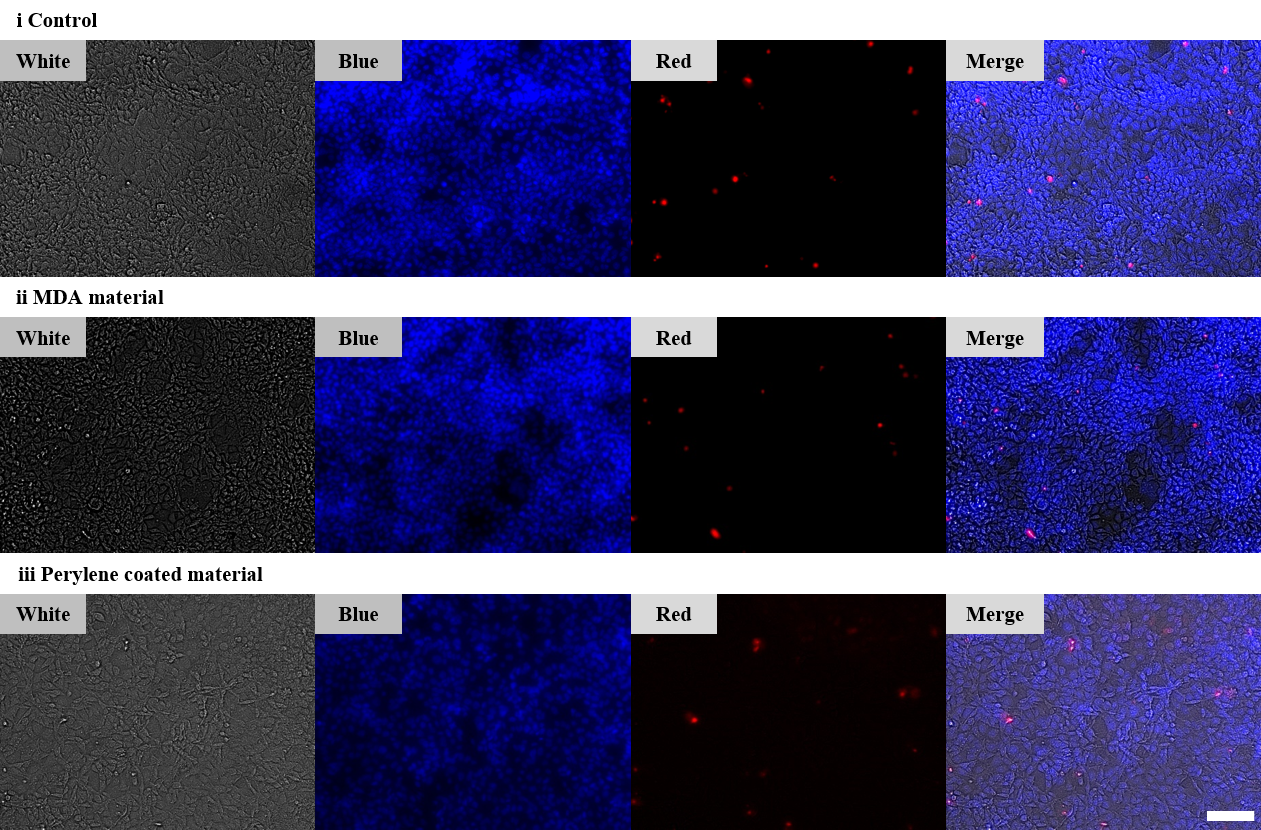


(a)


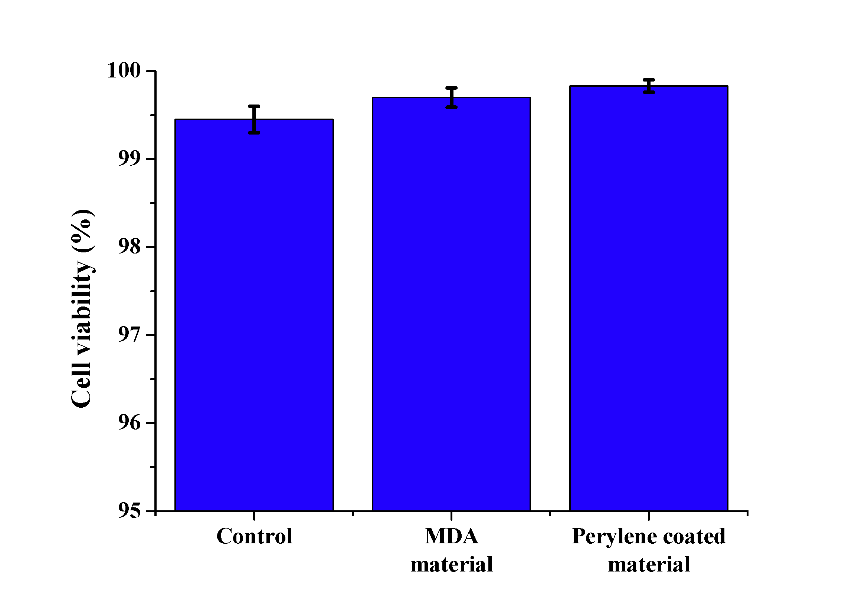


(b)

**Figure S4. Biocompatibility of the MDA material.** a) Bright-field and fluorescent micrographs of HCT cells after culture on MDA materials for 1 day. i) Control, ii) uncoated, and iii) parylene-coated MDA material. Hoechst 33342 (blue) and propidium iodide (red) were used to stain nuclei and dead cells, respectively. b) Cell viability relative to the control. Scale bar is 100 µm.

**Video Clips**

Video S1. Horizontal locomotion of the MDA in a cylindrical channel.

Video S2. Vertical locomotion of the MDA in a cylindrical channel.

Video S3. Horizontal locomotion of the MDA in a fluid flow channel.

Video S4. Vertical locomotion of the MDA in a fluid flow channel.

Video S5. Manipulation of the MDA in a horizontal network.

Video S6. Manipulation of the MDA in a vertical network.

Video S7. Drilling test of the MDA in a vertical channel.

Video S8. Manipulation and drilling test of the MDA in a 3D phantom coronary artery.
